# Effect of collagen endometrial patch loaded with adipose-derived mesenchymal stem cells on endometrial regeneration in rats with a thin endometrium

**DOI:** 10.3389/fendo.2023.1287789

**Published:** 2023-11-28

**Authors:** Juyeon Hong, Hyojin Ahn, Soo Young Moon, Hyo Jin Kang, Kyong Wook Yi

**Affiliations:** ^1^ Department of Obstetrics and Gynecology, Korea University College of Medicine, Seoul, Republic of Korea; ^2^ Department of Biomedical Laboratory Science, Honam University, Gwangju, Republic of Korea

**Keywords:** ADSC, bioprinting, endometrial patch, regeneration, thin endometrium

## Abstract

**Background:**

This study aimed to investigate the effects of a collagen endometrial patch (EM patch) loaded with adipose-derived mesenchymal stem cells (ADSCs) on endometrial regeneration in a rat model with thin endometrium.

**Materials and methods:**

Thin endometrium was induced in female rats and divided into treatment groups as outlined: control, group 1(G1), local injection of ADSCs into the uterus, group 2 (G2), an EM patch without ADSCs, group 3 (G3), and an EM patch loaded with ADSCs, group 4 (G4). The rats were euthanized at either two weeks or four weeks after modeling and treatment followed by histological and biochemical analyses to examine the regenerative effects on the injured endometrium.

**Results:**

Transplantation of the ADSC-loaded EM patch significantly promoted endometrial proliferation and increased the luminal epithelial area. Two weeks after treatment, the mean number of von Villebrand factor (vWF)^+^ or cluster of differentiation (CD) 31^+^-stained blood vessels was significantly higher in G4 than in G1 and G2. The mRNA and protein expression levels of TGF-β and FGF2 were significantly upregulated in G4 compared to those in the control. G4 exhibited significantly increased LIF mRNA levels and immunoreactivity compared with the other groups at both two weeks and four weeks after treatment. Cell tracking after ADSCs treatment revealed the presence of a substantial number of ADSCs grafted in the uterine tissues of G4, whereas a low number of ADSCs that were focally clustered were present in G2.

**Conclusion:**

Transplantation of EM patches loaded with ADSCs resulted in the histological and biochemical restoration of an injured endometrium. The strategic integration of EM patches and ADSCs holds significant promise as an innovative therapeutic approach for effectively treating impaired endometrial conditions.

## 1 Introduction

The endometrium is a dynamic remodeling tissue that undergoes histological, biochemical, and molecular changes during the reproductive cycle of females (1). Endometrial physiology is primarily regulated by sex steroid hormones and provides an essential environment for embryo implantation and pregnancy. Structurally and/or functionally impaired endometrium such as intrauterine adhesion (Asherman syndrome) or persistently thin endometrium, negatively affects endometrial receptivity, leading to a reduction in implantation and pregnancy rates in fertility treatment (1–3). A thin endometrium indicates impaired proliferation and growth of the endometrial epithelium, which fails to respond to endogenous estrogen. Although there is a lack of standardized criteria for a thin endometrium, it is commonly defined as an endometrial thickness of less than 7–8 mm based on ultrasound measurements (4–6). This condition may result from physical (curettage and uterine surgery) and biochemical (infection and drugs) factors that cause trauma to the stem cell-rich endometrial basalis (7). Recent studies have suggested that reduced endometrial blood flow, coupled with subsequent poor vascular development and impaired angiogenesis, constitutes the critical mechanism underlying a thin endometrium (8). A few therapeutic options, including hormonal manipulation (high-dose estrogen) or adjuvants (aspirin, sildenafil, and granulocyte colony-stimulating factor), have been used for thin endometrium-associated infertility, but the results remain controversial (9–14).

Stem cell therapy is a promising novel treatment approach for damaged and refractory endometria. Animal studies and a small series of clinical trials have demonstrated that adult stem cells derived from the bone marrow or adipose tissue can improve histological and functional restoration in the thin endometrium or uterine synechiae (15–19). Advancements in tissue engineering have highlighted the potential roles and utilization of biomaterials in regenerative medicine. A representative example is three-dimensional (3D) bioprinting technology. 3D bioprinting is an additive manufacturing process that involves the use of biomaterials, living cells, and active biomolecules to fabricate structures that mimic tissue properties. Bioprinting involves the addition of a non-toxic hydrogel that mimics the extracellular matrix environment to living cells to induce cell attachment, proliferation, and post-printing differentiation (20). Biomaterial scaffolds can provide structural components in various tissues and promote regeneration activity in combination with loaded stem cells, owing to their capacity for direct cellular differentiation, supporting the structurally and functional grafting of repopulating cells, thereby enhancing the therapeutic efficacy of stem cells (21).

The efficacy and feasibility of biomaterial scaffolds targeting impaired endometrial receptivity caused by damaged endometrial conditions require further study. In this study, we fabricated an endometrial patch (EM patch) based on a collagen scaffold using 3D bioprinting. To maximize the regeneration of the thin endometrium, adipose-derived stem cells (ADSCs) were bioprinted with a collagen bioink to produce an EM patch, and its therapeutic effect on repair and regeneration in a rat model with a thin endometrium was investigated.

## 2 Materials and methods

### 2.1 Fabrication of EM patch loaded with adipose-tissue derived mesenchymal stem cells using a 3D bioprinter

**Figure 1:**
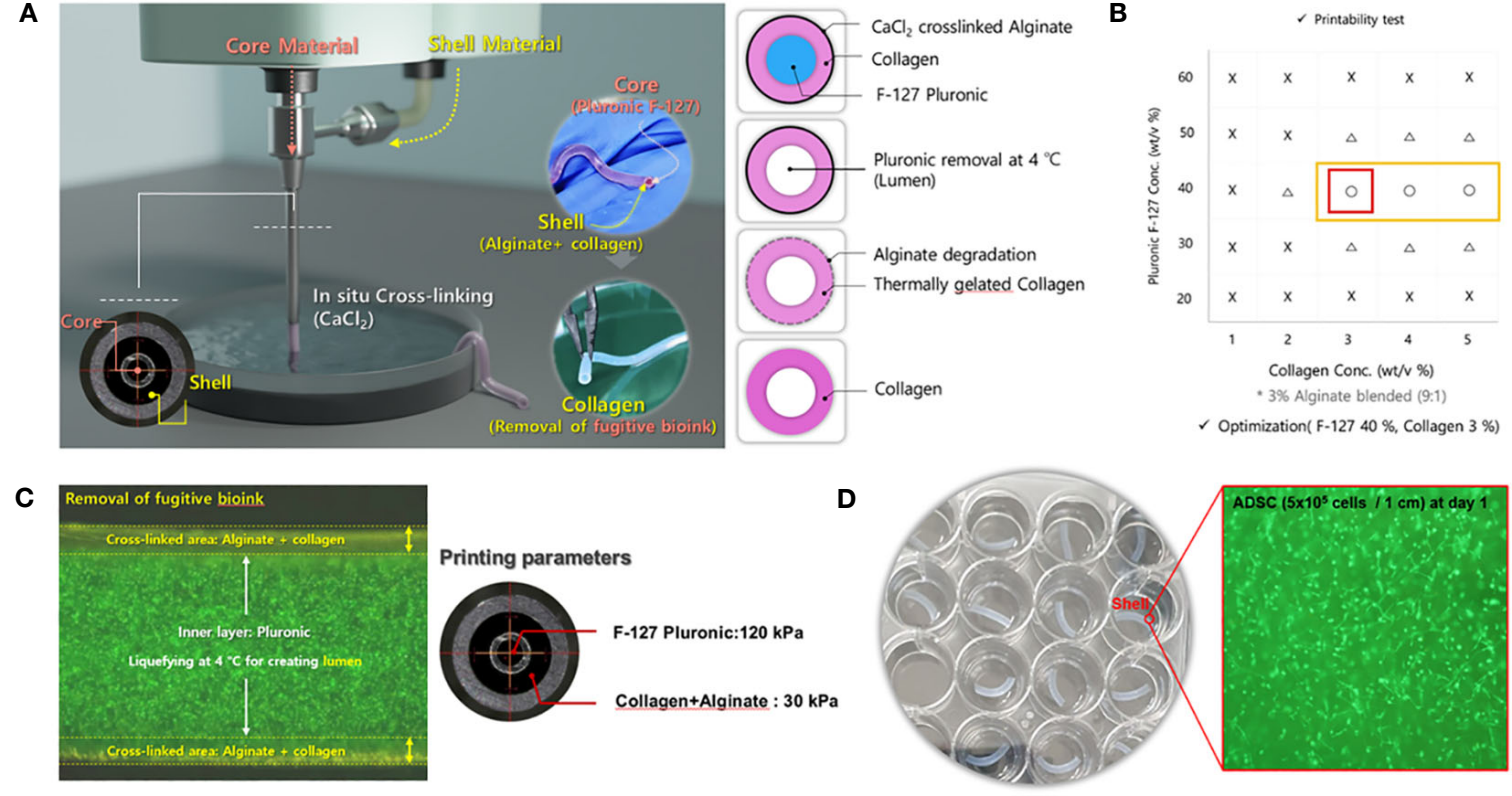
Experimental design and demonstration of the 3D-bioprinted endometrial (EM) patch. **(A)** Coprintability of each bioink combination (fugitive ink and cell-laden ink). **(B)** Schematic illustration of the bioprinting process for fabricating an EM patch; the fugitive ink (core) contains Pluronic, and the cell-laden ink contains atelocollagen, alginate, and adipose-derived mesenchymal stem cells (ADSCs). **(C)** *In-situ* cross-linking of alginate with atelocollagen and removal of the fugitive bioink and macroscale view of the co-axial nozzle and printing parameters. **(D)** Macroscale view of the bioprinted EM patch; the inset depicts the ADSC within the bioinks.

The fabrication process of the EM patch is shown in **Figure 1**. For bioink preparation, alginate (medium viscosity, ≥ 2,000 cP, A2033, Sigma-Aldrich, St. Louis, MO, USA) in Dulbecco’s phosphate-buffered saline (DPBS, Thermo Fisher Scientific, Waltham, MA, USA) was prepared and stirred for 3h at room temperature. Atelocollagen (Baobab Healthcare, Ansan, Korea) solution (pH 4.0) was neutralized with dibasic sodium phosphate (132 mM Na_2_HPO_4_) at a volume ratio of 1:1. Subsequently, the alginate and collagen solutions were mixed at a ratio of 1:9 for bioprinting. In addition, F-127 Pluronic (Sigma-Aldrich) was dissolved in DPBS at 40 wt% concentration to obtain a fugitive hydrogel. The rheological properties of the bioinks were measured by using a Kinexus Lab+ rheometer (NETZSCH, Selb, Bayern, Germany) equipped with a cone plate. To optimize coprintability, rheological analysis was conducted to evaluate a range of concentrations for the mixed bioinks. These bioinks comprised alginate and collagen solutions, spanning concentrations from 1 to 5 wt/v%, coupled with F-127 Pluronic spanning concentrations from 20 to 60 wt/v %. The rheological analysis was conducted at 37°C. Coprintable and unprintable conditions were determined by the shape of the printed specimen. Human adipose-derived mesenchymal stem cells (ADSCs) were purchased from CEFO (cat. CEFOTM hADMSC-001, Seoul, Korea) and 5×10^5^ cells/mL were resuspended in the mixed bioinks for bioprinting.

The cell tracker (DiO, D275), a lipophilic tracer, was purchased from Invitrogen (Waltham, MA, USA). The ADSC-laden EM patch was printed using coaxial nozzles (Ramé-Hart Instruments, USA) and a bioprinter (Root, Baobab Healthcare, Ansan, Korea). For cell-printing, 5×10^5^ cells/mL of ADSCs were resuspended into the mixed bioink and loaded into a syringe connected to a shell nozzle (13G). Simultaneously, the fugitive hydrogel (40 wt/v% F-127 Pluronic) was applied in a syringe connected to the core nozzle (18G). Subsequently, both bioinks were printed using pneumatic pressure (30 kPa and 120 kPa) into 150 mM CaCl_2_ to facilitate *in situ* cross-linking of alginate. Afterward, the samples were submerged in phosphate buffered saline (PBS) at 4°C for 1 min with shaking at 100 rpm to remove the fugitive hydrogel. Next, the samples were soaked in a complete medium and incubated at 37°C for 2h to facilitate the gelation of collagen in the bioink, which resulted in the formation of a tubular structure. The dimension of the EM patch was approximately 1.0 ± 0.2 mm (inner diameter) and 1.8 ± 0.2 mm (outer diameter). To analyzes cell viability, live cells were stained with calcein AM (green), and dead cells were stained with ethidium homodimer (red) according to the manufacturer’s instructions (LIVE/DEAD Viability/Cytotoxicity kit, Thermo Fisher Scientific, Waltham, MA, USA). Briefly, ADSC-laden (5×10^5^ cells/mL) EM patches were rinsed with PBS and incubated for 40 min. Following the incubation period, the cell-laden EM patches were rinsed again in PBS and were subjected to imaging using Olympus IX17 inverted microscope (Olympus corporation, Tokyo, Japan) employing the 488 and 543 nm channels.

### 2.2 Animals and modeling of thin endometrium

Female Sprague–Dawley rats were purchased from ORIENT BIO (Seongnam, Korea) and maintained according to the Association for Assessment and Accreditation of Laboratory Animal Care International System. All animal experiments conformed to the International Guide for the Care and Use of Laboratory Animals and were approved by the Institutional Animal Care and Use Committee of the Korea University College of Medicine (IACUC No. KOREA-2021-0188-C2).

**Figure 2:**
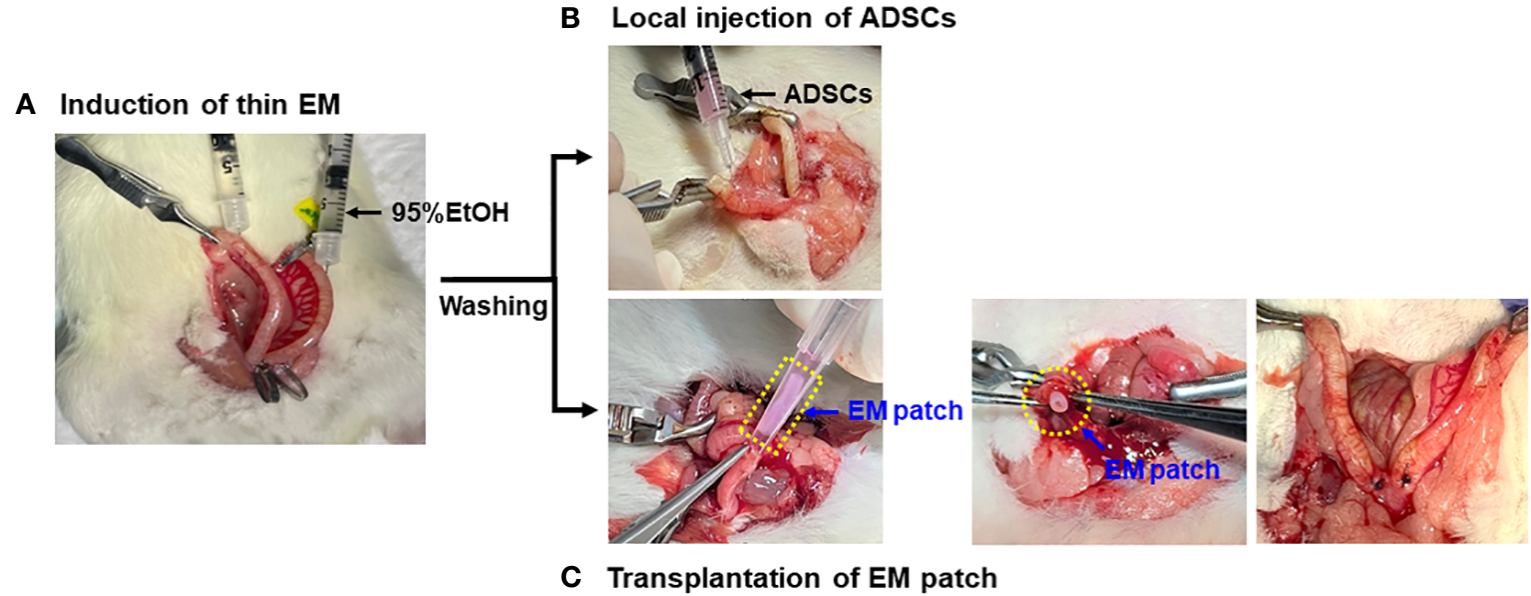
Modeling and administration of ADSCs. **(A)** Induction of thin endometrium with instillation of 95% ethanol. **(B)** Local injection of ADSCs into the uterine horns using 1mL syringe and 30G needles. **(C)** Transplantation of EM patch: a small incision was made at the proximal ends of bilateral uterine horns and the EM patch was gently placed into the uterine cavity, followed by the closure of incision by suture.

Nine-week-old female rats were anesthetized by isoflurane inhalation and the uterus was gently exposed through an abdominal incision. A thin endometrium was induced using 95% ethanol, as previously described (22). Briefly, vascular hemostatic clips were applied at the proximal and distal ends of the bilateral uterine horns and then 50 µL of 95% anhydrous ethanol was instilled into each uterine cavity using a 1mL tuberculin syringe equipped with a 30G needle. The clips were removed after five minutes and the uterine cavity was flushed with sterile PBS to remove any residual ethanol (**Figure 2A**). The rats induced with thin EM in the bilateral uterine horns were randomly divided into four groups (n=6 for each group) based on the mode of treatment: Group 1 (G1), control (no treatment); Group 2 (G2), local injection of ADSCs (5x10^5^ cells/50uL for each uterine horn) using a 1 mL syringe and needle immediately after thin EM modeling (**Figure 2B**); Group 3 (G3), transplantation of EM patch without ADSCs; and Group 4 (G4), transplantation of EM patch loaded with ADSCs (5x10^5^ cells per a patch). The procedure for the EM patch transplantation is shown in **Figure 2C**. The rats were euthanized at two weeks and four weeks after thin EM modeling and treatment, and uterine tissues (12 samples per group) were collected for histological and biochemical analyses.

### 2.3 Tracking of ADSCs in uterine tissues following local injection or EM patch transplantation

Twenty-five mg of (3,3’-dioctadecyloxacarbocyanine perchlorate) DiO and 5 mg of octadecylamine were mixed in 0.5 mL of chloroform and heated at 56 °C for 5 min. Then, 1 mL of methanol was added, and the mixture was centrifuged at 13,000 rpm for 5 min. The supernatant was removed, and the pellet was dried. Finally, a stock solution was prepared by dissolving the dried pellet in 1.0 mL of dimethyl sulfoxide (DMSO, Sigma-Aldrich). The stock solution was diluted 100 times in the ADSCs suspension and centrifuged at 6,900 g for 5 min. Thereafter, the cell pellet was washed twice in 0.15M Tris buffer. The labeled ADSC (5x10^5^cells/50uL) was locally injected into the endometrium in G2 or loaded to the EM patch which was transplanted into the endometrium in G4. Uterine tissues were obtained after two or four weeks of treatment and labelled ADSCs were visualized using a fluorescence microscope (Carl Zeiss Axio Vision4, Carl Zeiss MicroImaging GmbH).

### 2.4 RNA extraction and quantitative real-time polymerase chain reaction

RNA was extracted from the uterine horns using TRIzol reagent (Molecular Research Center, Cincinnati, OH, USA) according to the manufacturer’s instructions. The concentration and purity of total RNA were measured using a Nano drop 2000c spectrophotometer (Thermo Fisher Scientific, Waltham, MA, USA) at absorbances of 260, 280 and 230 nm. First-strand cDNA was synthesized using a TOPscript™ RT DryMIX (Enzynomics, Daejeon, Korea) kit. Reverse transcription was performed using the program settings provided by the manufacturer.

The mRNA levels of TGF-β, FGF2, and LIF were determined using qRT-PCR. qRT-PCR was performed using the Power SYBR™ Green PCR Master Mix (Applied Biosystems™ Thermo Fisher Scientific, Waltham, MA, USA) on a QuantStudio™ 5 Real-Time PCR System (Applied Biosystems™ Thermo Fisher Scientific). The forward and reverse primers used in the study were as follows: 5’-CGTGGAAATCAATGGGATCAG-3’ and 5’-CAGGAAGGGTCGGTTCATGT-3’ for TGF-b; 5’-GAACCGGTACCTGGCTATGA-3’ and 5’-CCGTTTTGGATCCGAGTTTA-3’ for FGF2; 5’-ACCAGATCAAGAGTCAACTG-3’ and 5’-CCTTGAGCTGTGTAATAGGA-3’ for LIF; 5’-GCTGTGTTGTCCCTGTATGC-3’ and 5’-GAGCGCGTAACCCTCATAGA-3’ for β-actin.

In PCR reactions, the protocol included an initial denaturation for 10 min at 95°C followed by 40 cycles comprising denaturation at 95°C for 15 sec, annealing at 52°C for 30 sec, and extension at 72°C for 30 sec. Subsequently, a dissociation curve analysis was performed. Gene expression was normalized to the expression of β-actin as an internal control, and the relative mRNA expression was calculated using the comparative cycle threshold method.

### 2.5 Histopathological assessment

Uterine tissues from sacrificed rats were harvested and preserved in 10% formalin. Following fixation, the tissues were embedded in paraffin and subjected to routine processing protocols. Subsequently, the specimens were sliced into sections measuring 4–5 µm in thickness. The sections were deparaffinized in xylene at room temperature and stained with hematoxylin and eosin (H&E) (Cancer Diagnostics Inc., Durham, NC, USA) according to the manufacturer’s instructions. Masson’s trichrome staining (BBC Biochemical, Mount Vernon, WA, USA) was performed according to the manufacturer’s instruction. Briefly, deparaffinized sections were fixed in Bouin’s solution for 1 h at 56°C and stained with ClearView Iron Hematoxylin working solution for 10 min. The tissues were then stained with Biebrich scarlet-acid fuchsin solution (2 min), phosphomolybdic-phosphotungstic acid solution (10 min), aniline blue solution (3 min) and 1% acetic acid solution (2 min). The extracellular matrix (ECM), collagen, and other connective tissue elements were stained blue and smooth muscle was stained red. Tissue sections were imaged using a slide scanner (3DHISTECH Ltd., Budapest, Hungary).

### 2.6 Immunohistochemistry and immunofluorescence analysis

Immunohistochemistry (IHC) was performed using the diaminobenzidine-based staining technique using the GBI Polink-2 HRP kit (Golden Bridge International Inc., Bothell, WA, USA). Briefly, antigen retrieval was performed with 0.01 M sodium citrate buffer (pH 6.0) using a water bath after deparaffinization and rehydration of sections. These sections were then incubated for 10 min in a peroxide blocking buffer (Scytek Laboratories, Inc., West Logan, UT, USA). Subsequently, the sections were incubated with a rat LIF antibody (1:50, Bioss Antibodies, USA) followed by the Polink-2 Plus HRP anti-rat DAB Detection kit (Golden Bridge International Inc., Bothell, WA, USA) according to the manufacturer’s instructions. The sections were stained with diaminobenzidine (Golden Bridge International, Inc., USA), counterstained with Mayer’s hematoxylin (Scytek Laboratories, Inc., West Logan, UT, USA), and dehydrated with alcohol. After clearing with xylene and covering with a cover glass, images were obtained using a slide scanner. LIF expression was quantified using ImageJ software, and the stained area was measured as percentage and then compared among the groups.

Immunofluorescence (IF) was performed using a GBI Polink-2 HRP kit (Golden Bridge International Inc., Bothell, WA, USA). Briefly, the sections were deparaffinized in xylene at room temperature and rehydrated in a graded series of ethanol. Heat-induced epitope retrieval was conducted using Tris-EDTA, pH 8.0 (Scytek Laboratories, Inc., West Logan, UT, USA). Following this, the sections were incubated overnight at 4°C with primary antibodies, including vWF (1:200, EMD Millipore, Temecula, CA, USA) and CD31 (1:500, Thermo Fisher Scientific, Waltham, MA, USA). Subsequently, the sections were incubated with secondary antibodies, Alexa 594 and 488 (Thermo Fisher Scientific) for 2 h at room temperature. They were mounted with a (4’,6-diamidino-2-phenylindole) DAPI-containing mounting medium (Golden Bridge International Inc., Bothell, WA, USA) and observed using a Carl Zeiss AxioVision 4 (Carl Zeiss MicroImaging GmbH). For the quantification of CD31- or vWF-positive vessels, at least five different spots in each section were counted in a high magnification field (×400), and the sum of the positive vessels was compared among the experimental groups.

### 2.7 Western blot analyses

Uterine tissues were homogenized using PROPREP lysis buffer (Intron, Seoul, Korea) and proteins were quantified using a BCA Protein Assay Kit (Thermo Fisher Scientific, Waltham, MA, USA). Briefly, samples with equal concentrations of protein were mixed with 5× sample buffer (ELIPIS BIOTECH, Namyang, Korea), heated at 95°C for 10 min, and electrophoresed on a 10% sodium dodecyl sulfate-polyacrylamide gel. (SDS-PAGE). Proteins were transferred onto polyvinylidene fluoride membranes (ATTO, Tokyo, Japan) using Tris-glycine transfer buffer (ATTO). Following this, the membranes were subjected to blocking with 5% skim milk (BD Biosciences) for 1 h at room temperature. The membranes were then incubated overnight at 4°C with primary antibodies, including anti-FGF2 (1:500, Santa Cruz Biotechnology, Dallas, Texas, USA), anti-TGF-β (1:1000, Abcam, Cambridge, UK), and anti-β-actin (1:1000, Santa Cruz Biotechnology, Dallas, Texas, USA). Subsequently, the membranes were incubated with the secondary antibody for 2 h, either an anti-mouse antibody (1:10000, Cell Signaling Technology, Danvers, MA, USA) or an anti-rabbit antibody (1:10000, Cell Signaling Technology, Danvers, MA, USA) was used. Following this, the membranes were washed and the proteins were detected using the West-Q Chemiluminescent Substrate Plus kit (GenDEPOT Inc., Barker, TX, USA). The intensity of the protein bands was determined using Multi Gauge software (version 3.0; Fuji Photo Film, Tokyo, Japan). The relative densities were expressed as a ratio of the control values. All reactions were performed in triplicates.

### 2.8 Statistical analyses

One-way analysis of variance with *post-hoc* least significant difference tests was used to assess the differences in the continuous variables among the groups. Statistical analyses were performed using the Statistical Package for Social Science, version 20.0 software (SPSS, Chicago, IL) and *P*-value < 0.05 were considered to be statistically significant.

## 3 Results

### 3.1 Fabrication of EM patch using a coaxial nozzle

In tissue engineering, researchers often use sacrificial materials to create microchannels or coaxial nozzles, facilitating the *in situ* formation of blood vessels and preventing ischemic conditions (23, 24). Similarly, an EM patch was fabricated using a coaxial nozzle to form a tubular shape. First, a Pluronic F-127 core material (i.e., fugitive material), which can flow at 4°C but not at 37°C, and a shell material consisting of alginate and atelocollagen were used. Both bioinks were printed into a CaCl2 solution using a coaxial nozzle (inner needle 23G, outer needle 17G, Ramé-hart) equipped with a 3D bioprinter (Root 1, Baobab Healthcare). Alginate was simultaneously cross-linked and infused with collagen and Pluronic. Subsequently, Pluronic was removed at 4°C and incubated at 37°C within a cultured medium. Finally, alginate was gradually degraded and collagen was thermally gelated, and vice versa (**Figure 1A**). Next, we assessed the coprintability of each bioink combination. The coprintable concentration of the shell bioink was in the range of 3–5 wt/v %, and that of the core bioink was 40 wt/v % (**Figure 1B**). To remove fugitive bioink, Pluronic was blended with green fluorescent particles and simultaneously liquefied at 4°C. The EM patches were printed at 120 kPa (core) and 30 kPa (shell) (**Figure 1C**). The resulting sample was cut at 1 cm intervals, and the inner and outer sizes were 1.0 ± 0.2 mm (Ø) and 1.8 ± 0.2 mm (Ø), respectively. The cell-laden EM patch contained ADSCs (5 × 105 cells/1 cm), and cell viability was over 90% on day 1 (**Figure 1D**).

### 3.2 Tracking of ADCSs in groups subjected to local injection or groups receiving ADSC-loaded EM patch transplantation

**Figure 3:**
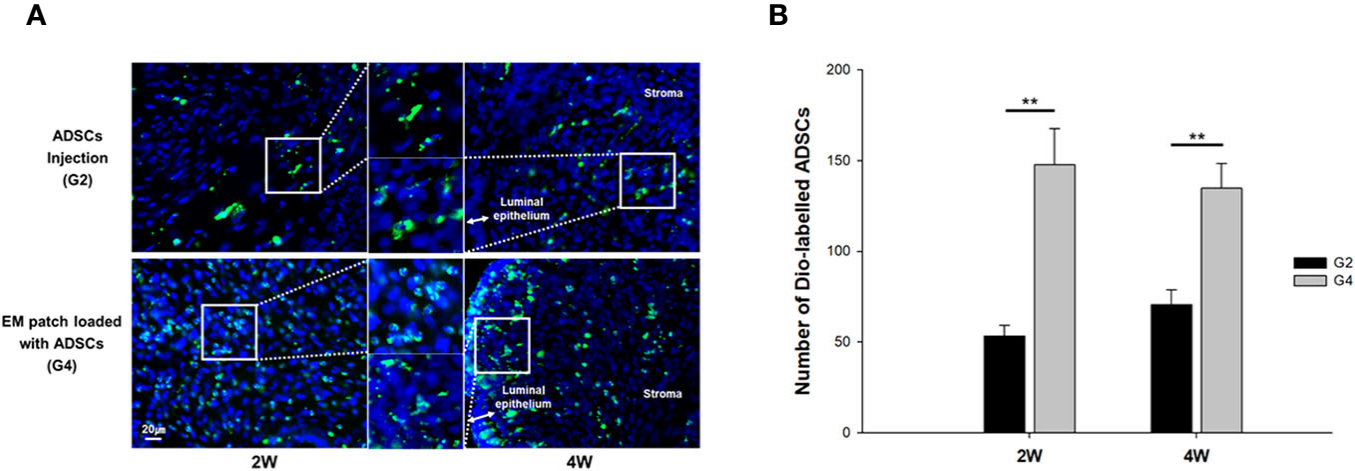
Fluorescence micrographs of (3,3’-dioctadecyloxacarbocyanine perchlorate) DiO -labelled ADSCs in uterine tissues at two and four weeks after modeling and treatment. **(A)** Local injection of ADSCs into the uterine horns, group 2 (G2) (upper), and transplantation of ADSC-loaded EM patch, group 4 (G4) (lower). **(B)** Comparison of the number of Dio-labelled ADSCs in uterine tissues at two and four weeks. ^**^ *P* < 0.01.

We performed cell tracking analysis to examine the number and patterns of ADSCs in the groups treated with local injection (G2) or transplantation of EM patches loaded with ADSCs (G4). The rats were sacrificed at two and four weeks post-treatment for the tracking of DiO-labelled ADSCs. The labelled ADSCs were visualized within the luminal epithelia and uterine stroma of both groups (**Figure 3A**). The transplantation of ADSC-loaded EM patches resulted in a greater number of DiO-labelled ADSCs grafts compared to that of the local injection of ADSCs at both two and four weeks (*P* = 0.01, *P* = 0.01, respectively) (**Figure 3B**); the local injection of ADSCs yielded fewer cells that were focally clustered in the uterine tissues.

### 3.3 Histological analyses of the uterine structure

**Figure 4:**
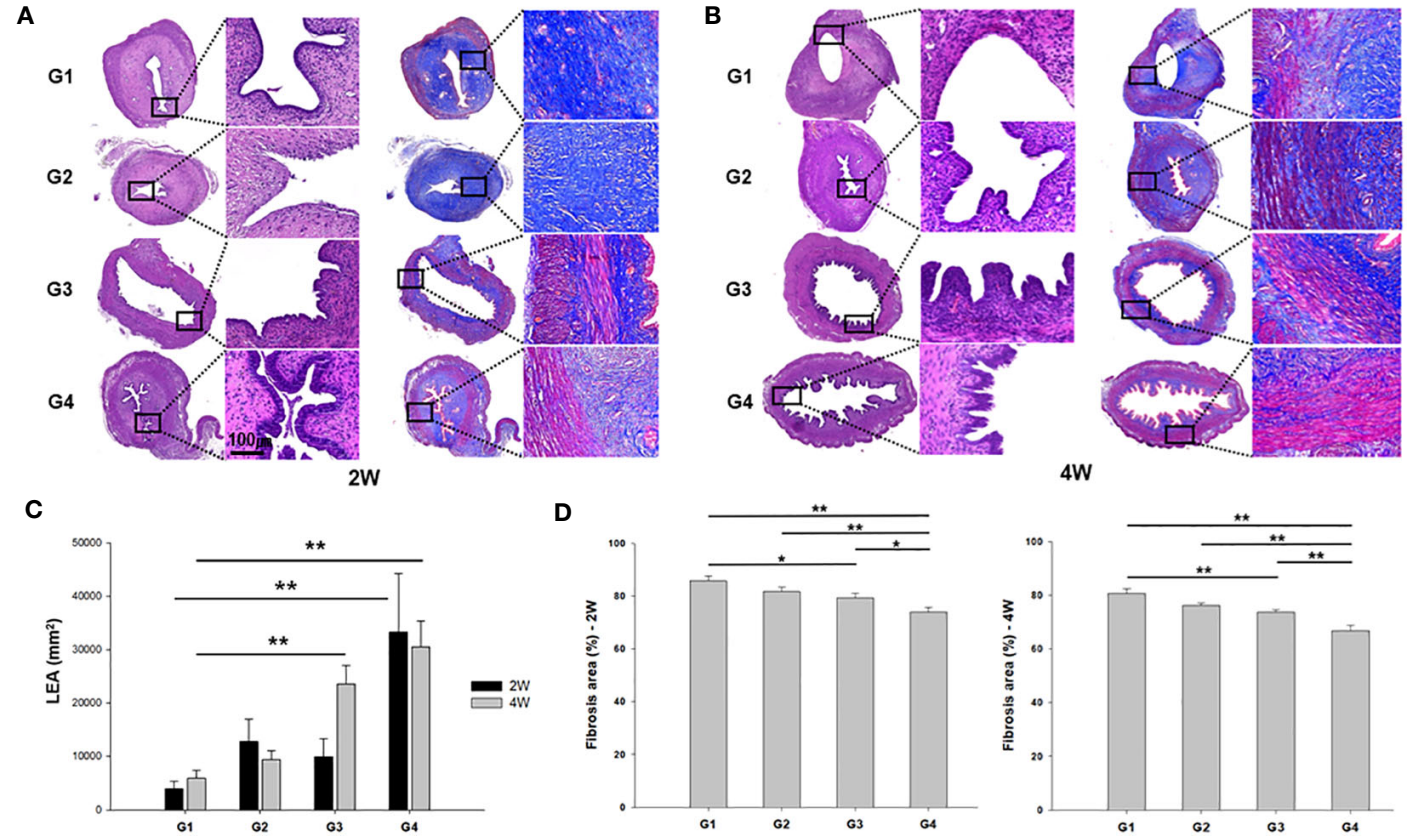
Representative images of hematoxylin and eosin (H&E) and Masson’s trichrome staining of uterine sections from each group. **(A)** two weeks (left) and **(B)** four weeks (right) after modeling and treatment. **(C)** Luminal epithelial area (LEA) and **(D)** fibrosis area (%) were measured in uterine sections and compared among all groups. ^*^ *P* < 0.05, ^**^ *P* < 0.01.

Hematoxylin and eosin (H&E)- and Masson’s trichrome-stained images of the uterine horns in all groups are shown in **Figure 4**. At two weeks after treatment, the endometrial epithelium was much more proliferative and extensively branched in G4 in comparison to the other groups, whereas it remained thin and dilated in G1 (**Figure 4A**). Modest proliferative change was observed in G3 rats. Four weeks after treatment, the endometrium showed further proliferative changes and less fibrosis in G3 and G4 groups than the G1 group (**Figure 4B**).

Quantitative analysis of luminal epithelial area (LEA) exhibited a significant increase in G4 compared to G1 at both the two weeks (*P* = 0.006) and four weeks (*P* < 0.001) post-treatment (**Figure 4C**). Furthermore, G3 displayed a significantly larger LEA than G1 in the 4 week-model (*P* < 0.01). Quantification of the fibrosis areas by Masson’s trichrome staining revealed increased collagen fiber deposition in the stroma in G1 and G2, and a significant reduction was observed in fibrosis in the uterine horns of G4 compared to those of all other groups at both two and four weeks after modeling and treatment (**Figure 4D**).

### 3.4 Measurement of blood vessels by colocalization of vWF and CD31

**Figure 5:**
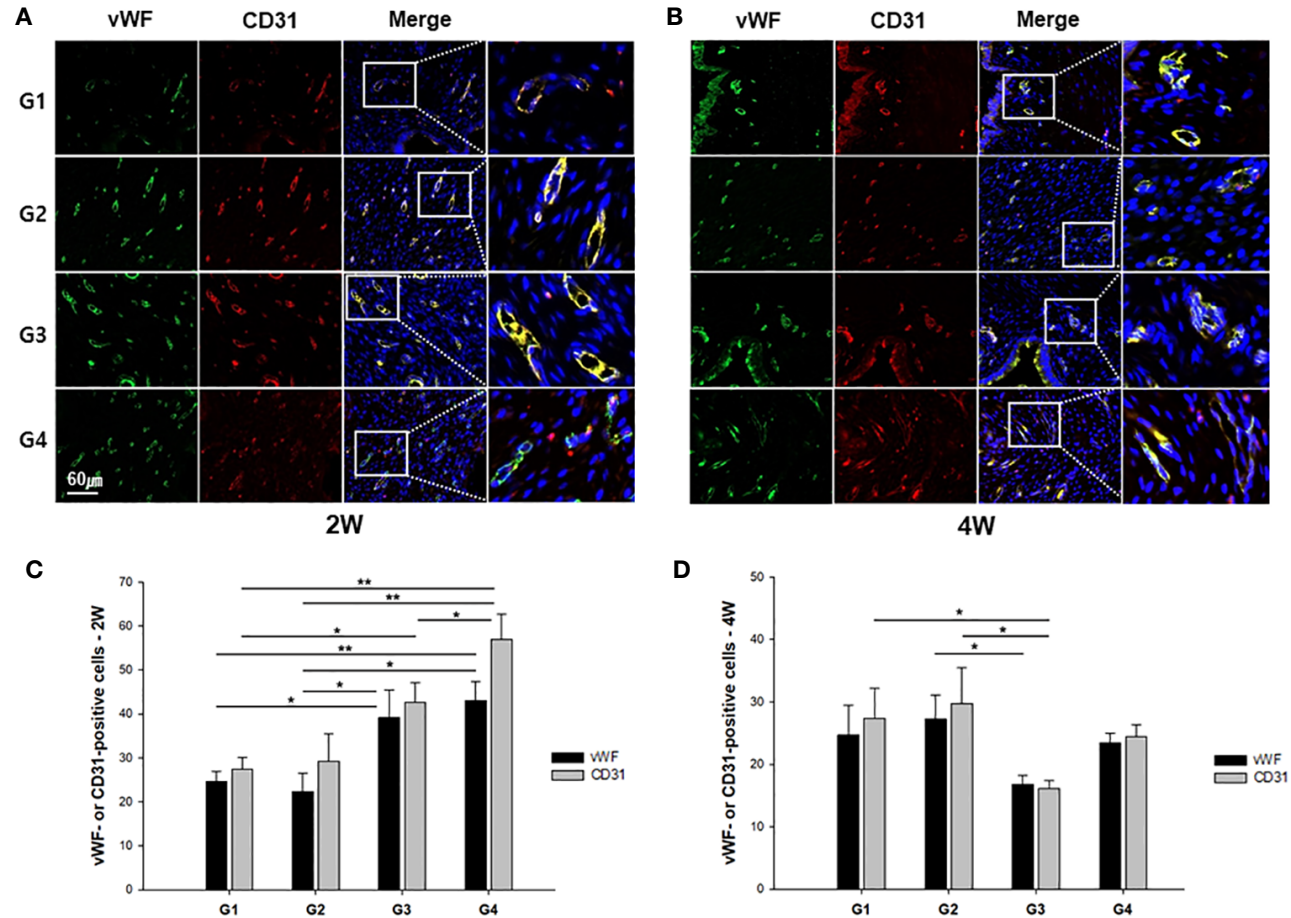
Representative immunofluorescence images of uterine sections from each group. The sections were double stained with anti-vWF (green) or anti-CD31 (red) antibody at **(A)** two weeks or **(B)** four weeks after modeling and treatment. The quantified number of vWF- or CD31-stained vessels was compared among the groups at **(C)** 2 weeks and **(D)** 4 weeks. ^*^ *P* < 0.05, ^**^ *P* < 0.01.

IF staining with anti-vWF and anti-CD31 antibodies was performed to examine blood vessel formation in the uterine sections of all groups, as shown in **Figures 5A**, **B**. In quantification of vWF-positive cells at two-week models, G4 showed a statistically greater number of vWF^+^ blood vessels compared to both G1 and G2 (*P* < 0.01 and *P* < 0.01, respectively) (**Figure 5C**). In addition, G3 demonstrated more vWF^+^ blood vessels than G1 and G2 (*P* = 0.028 and *P* = 0.031, respectively). The average number of CD31^+^ vessels was significantly greater in G4 in comparison to G1, G2, and G3 (*P* < 0.01, *P* < 0.01, and *P* = 0.038, respectively) at two weeks post-treatment. Additionally, G3 displayed a significantly increased number of CD31^+^ vessels in comparison to G1 (*P* = 0.029). **Figure 5D** illustrates vWF^+^ or CD31^+^ cells at four weeks after treatment, presenting that there were more vWF^+^ vessels in G2 in comparison to G3 (*P* = 0.021). The mean number of CD^+^ vessels was significantly higher in both G1 and G2 as compared to G3 (*P* = 0.029 and *P* = 0.013, respectively).

### 3.5 mRNA and protein expression of TGF-β and FGF2

**Figure 6:**
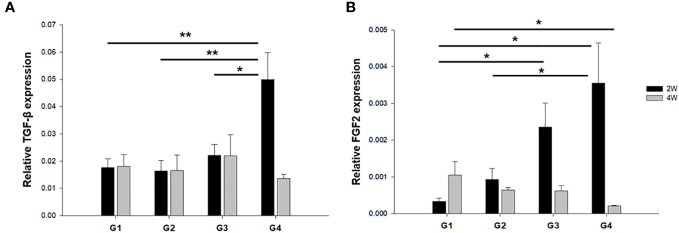
The mRNA expression of TGF-β and FGF2 in uterine horns of all groups at **(A)** two weeks and **(B)** four weeks after modeling and treatment. ^*^ *P* < 0.05, ^**^ *P* < 0.01.

We evaluated the expression of cytokines and growth factors that exert multifunctional capacities in regulating cell growth and proliferation, the immune system, and the repair or regeneration of damaged tissues (25–28). The mRNA expression of TGF-β was significantly upregulated in G4 compared to the other groups at 2 weeks post-treatment (**Figure 6A**). However, there was no significant difference between the groups at 4 weeks after treatment. FGF2 mRNA levels were also significantly upregulated in G4 compared with those in G1 and G2 in the uterine sections 2 weeks after treatment (*P* < 0.01 and *P* = 0.014, respectively (**Figure 6B**). However, increased FGF2 mRNA expression levels were observed in G1 compared to G4 (*P* = 0.02) at 4 weeks post-treatment.

**Figure 7:**
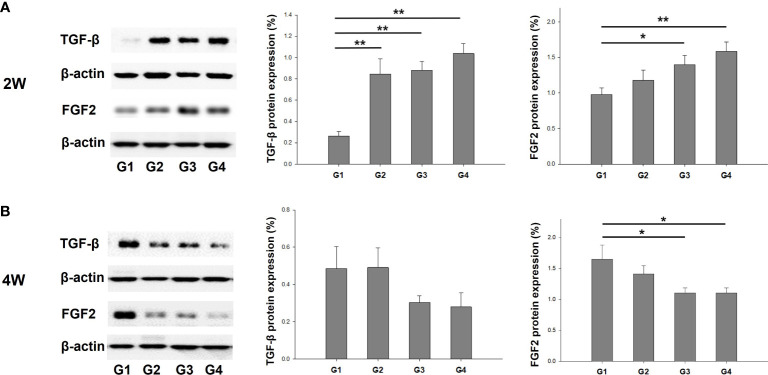
Western blot analyses of TGF-β and FGF2 expression normalized to β-actin as internal control in uterine tissues. Corresponding quantification values were compared among the groups at each time point of **(A)** two weeks and **(B)** four weeks after modeling and treatment. ^*^ *P* < 0.05, ^**^ *P* < 0.01.

The protein expression of TGF-β and FGF2 was quantified through western blotting and normalized to total tissue protein levels approximated using β-actin. This normalization was carried out to facilitate a comparison among the groups. The results showed that TGF-β protein expression was significantly increased in G2, G3, and G4 compared to G1 (*P* < 0.01, *P* < 0.01, and *P* < 0.01, respectively) at 2 weeks post-treatment (**Figure 7A**). However, there was no significant difference in TGF-β protein expression among the groups at 4 weeks after treatment (**Figure 7B**). FGF2 protein expression was higher in G3 and G4 than in G1 (*P* = 0.029 and *P* < 0.01, respectively) at two-weeks-model (**Figure 7A**), whereas there was a significant increase in FGF2 protein expression in G1 compared to that in G3 and G4 (*P* = 0.017 and *P* = 0.038, respectively) in 4 weeks-model (**Figure 7B**).

### 3.6 Analysis of LIF expression using IHC and qRT-PCR

**Figure 8:**
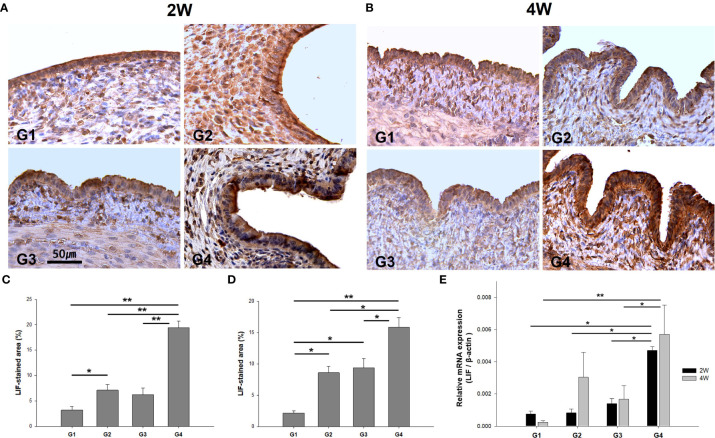
The immunostaining and mRNA expression of LIF in uterine sections. Representative images of immunohistochemistry of anti-LIF antibody in uterine sections at **(A)** two weeks and **(B)** four weeks. Quantification by H-score at uterine tissues was obtained at **(C)** two weeks and **(D)** four weeks post-treatment. **(E)** Comparison of LIF mRNA levels among the groups. ^*^ *P* < 0.05, ^**^ *P* < 0.01.

The immunoreactivity of LIF, an endometrial receptivity marker, was detected in the luminal and glandular epithelia (**Figures 8A, B**). It was observed that the H-score for LIF expression was significantly higher in G4 than in G1, G2, and G3 at 2 weeks (**Figure 8C**) and 4 weeks (**Figure 8D**) post-treatment. Compared to G1, G2, and G3 (*P* = 0.023, *P* = 0.017, and *P* = 0.038, respectively), LIF mRNA expression levels were upregulated in G4 at 2 weeks post-treatment (**Figure 8E**). Additionally, LIF mRNA expression was significantly elevated in G4 than in G1 and G3 (*P* < 0.01 and *P* = 0.042, respectively), as observed from the 4-week-old-specimens.

## 4 Discussion

A thin endometrium is an unresolved pathologic condition associated with a lower implantation rate and increased miscarriage; however, a definitive treatment modality has not yet been established. To improve the endometrial receptivity caused by damaged endometrium, research has focused on the potential role of stem cell therapy in endometrial repair and restoration. Mesenchymal stem cells (MSCs) are adult stem cells that can be isolated from various tissues such as the bone marrow, endometrial tissue, or adipose tissue. They serve as therapeutic agents in the field of regenerative medicine based on their self-renewal capacity and differentiation potentials (29–32).

In this study, we fabricated a collagen EM patch designed with a tubular structure suitable for the damaged uterine cavity in the rats using a 3D-bioprinter. The transplantation of EM patches loaded with ADSCs effectively restored the proliferation of endometrial epithelia and reduced fibrosis in the stroma of damaged uterus. In addition, treatment with ADSC-loaded EM patches induced a significant upregulation of TGF-β and FGF2 gene expression compared to the other treatment groups. TGF-β serves multifaceted roles in wound healing and tissue repair by enhancing matrix protein synthesis and deposition of vascular smooth muscle and endothelial cells. Moreover, TGF-β exerts immunomodulatory effects, including inhibition of chemotactic migration and proliferation of neutrophils and macrophages (28, 33, 34). FGF2 functions in various cellular events that facilitate wound repair and healing by promoting cell survival, proliferation, migration, and angiogenesis (35, 36). Several studies have reported the paracrine effects of ADSCs on tissue repair and wound healing via the production of FGF2 and TGF-β in various injured tissues (37–39). Consequently, transplantation of ADSC-loaded EM patch could potentially promote the secretion of TGF-β and FGF2, which exert modulatory effects and could have contributed to the restoration of the uterine structures in our study.

We found that the EM patch without ADSCs (G3) demonstrated partial improvements in certain histological and biochemical parameters, and vessels formation in comparison to those of the untreated control. The ECM surrounding the tissues plays a critical role in cell proliferation and differentiation. It creates a framework and provides various essential components for cell growth (40, 41). Collagen is one of the basic structural elements of the ECM that provides mechanical support and regulates cell behaviors (42). Consequently, it can be speculated that the acellular collagen EM patch in our study was able to initiate partial recovery of wounded endometrium; however, these effects were less prominent than those induced by the EM patch loaded with ADSCs.

In previous studies of stem cell therapy in endometrial injury models, several routes of stem cell administration, such as intrauterine injection or systemic injection through the tail vein have been reported (16, 19, 43–45). More recently, there has been emerging interest in strategies for stem cell therapy to facilitate storage and transportation and further enhance the survival and differentiation of transplanted stem cells (46). The difficulty of long-term engraftment of transplanted cells in target tissues has been discussed as a potential factor affecting the efficacy of stem cell therapy (47, 48). The advantages of biomaterial scaffolds in combination with stem cells include an extension of the duration of contact with damaged tissue, thereby enhancing the therapeutic efficacy of stem cells by maintaining their viability (40, 49). Cell tracking analysis in our study revealed that transplantation of EM patches loaded with ADSCs induced a greater number of ADSCs grafts with spatial distribution in the uterine tissues compared to the treatment involving local injection of ADSCs, where fewer cells were focally clustered in distribution. These results were consistent in the uterine tissues at both two weeks and four weeks post-treatment. These findings suggest that the utilization of a 3D-printed EM patch loaded with ADSCs represents a more efficacious approach to delivering ADSCs. This is achieved by establishing contact with the damaged endometrial surface. Furthermore, the cell grafts have the potential to be sustained until the degradation of the biomaterial.

The strength of our study lies in its design, where experiments were conducted within two distinct time frames, a span of two weeks and four weeks following modeling and treatment. This comprehensive approach enables us to discern and analyze time-dependent effects of various treatment groups on the regeneration of uterine structures. Histological findings indicating restoration of uterine structures, increased micro-vessel density, and favorable changes in biochemical and endometrial receptivity markers were noted earlier in G4 than in G2 or G3, and were evident even 2 weeks after the treatment. However, some of the results, including blood vessel volume and expression of cytokines or growth factors, did not show significant differences among the groups in the four-week model. A plausible interpretation of these findings is that the cellular events, such as angiogenic and immunomodulatory responses facilitated by the EM patch with ADSCs, exhibited early and rapid dynamics; this is supported by the findings observed at two weeks post-treatment. Hence, these effects seemed to diminish and wane as the study progressed to the four-week-post-treatment stage.

IF (vWF and CD31) analysis demonstrated that neovascularization was significantly enhanced in G3 and G4 relative to G1. At two weeks after treatment, the mRNA expression of FGF2 was significantly increased in G3 and G4 compared to G1, but TGF-β exhibited a significant increase only in G4. Although the differential expression of TGF-β and FGF2 is challenging to definitely interpret considering the myriad cellular events inherent to the wound healing process, FGF2 is one of the strongest angiogenic inducers and is released from the ECM as an initial process in wound healing at acutely damaged tissues (50–52). Therefore, the possible explanation for the FGF2 mRNA increase in G3 not only in G4 at two weeks suggests an earlier upregulation of FGF2 in response to EM patch (with or without ADSCs), which might be a crucial contributing factor to the vessel formation, as evident from increased presence of vWF^+^ and CD31^+^ cells in both G3 and G4. The results of the mRNA and protein expression of FGF2 at four weeks after treatment showed that G1 exhibited significantly enhanced levels of FGF2 compared to those of G3 or G4, which might be explained by the fact that certain delayed healing processes may occur in response to endometrial injury in the untreated controls.

A few studies have reported promising results utilizing biomimetic scaffolds combined with adult stem cells derived from the bone marrow or umbilical cord for the regeneration of endometrial injury models (53–55). Only a few studies have investigated the therapeutic efficacy of ADSCs (with local injection) in injured endometrium; however, they demonstrated favorable outcomes, since this treatment was associated with reduced inflammation and fibrosis, stimulated angiogenic factors, and improved the histological regeneration of uterine structures in Asherman syndrome models (18, 19). ADSCs have the advantages of abundant reserves *in vivo*, are easily obtained, and have a greater capacity for proliferation and secretion than bone marrow-derived stem cells (46, 48, 56, 57). Consequently, ADSCs stand out as a highly promising avenue, carrying significant weight in the arena of cell therapy for tissue regeneration (39, 46). Our EM patch, fabricated by optimizing printing parameters using a 3D-bioprinting device in combination with ADSCs not only confirmed the therapeutic potential of ADSCs on established thin endometrium models but also demonstrated the biological feasibility and benefits of the biomaterial scaffold for better transport and retention of stem cells in uterine tissues.

In conclusion, few therapeutic options are currently available in reproductive medicine for damaged thin endometrium and related infertility issues. Transplantation of ADSC-loaded EM patches effectively restored the histological architecture of the uterus and induced a favorable profile for the modulation of cytokines, growth factors, and endometrial receptivity markers. Therefore, the integration of 3D-printed scaffolds incorporating ADSCs presents itself as a highly promising therapeutic strategy in the field of cell therapy. This approach not only offers a prospective alternative to classical stem cell therapy, but also addresses the specific challenges posed by injured endometrial conditions and resulting infertility.

## Data availability statement

The original contributions presented in the study are included in the article/supplementary material. Further inquiries can be directed to the corresponding authors.

## Ethics statement

The animal study was approved by Institutional Animal Care and Use Committee of the Korea University College of Medicine. The study was conducted in accordance with the local legislation and institutional requirements.

## Author contributions

JH: Investigation, Methodology, Writing – original draft, Writing – review & editing. HA: Data curation, Methodology, Validation, Writing – original draft, Formal Analysis, Writing – review & editing. SYM: Investigation, Validation, Writing – original draft, Methodology, Writing – review & editing. HJK: Conceptualization, Funding acquisition, Investigation, Methodology, Supervision, Validation, Visualization, Writing – original draft, Writing – review & editing, Formal Analysis. KWY: Conceptualization, Formal Analysis, Funding acquisition, Investigation, Methodology, Software, Supervision, Validation, Visualization, Writing – original draft, Writing – review & editing.
